# Study on the Mechanism of Acori Graminei Rhizoma in the Treatment of Alzheimer's Disease Based on Network Pharmacology and Molecular Docking

**DOI:** 10.1155/2021/5418142

**Published:** 2021-12-24

**Authors:** Yi Kuan Du, Yue Xiao, Shao Min Zhong, Yi Xing Huang, Qian Wen Chen, Yu Qi Zhou, Jin Yan Guo, Chun Yang

**Affiliations:** ^1^Central Laboratory, Affiliated Dongguan Hospital, Southern Medical University, Dongguan, Guangdong, China 523059; ^2^Key Laboratory of Stem Cell and Regenerative Tissue Engineering, Guangdong Medical University Dongguan, China 523808

## Abstract

Alzheimer's disease is a common neurodegenerative disease in the elderly. This study explored the curative effect and possible mechanism of Acori graminei rhizoma on Alzheimer's disease. In this paper, 8 active components of Acori graminei rhizoma were collected by consulting literature and using the TCMSP database, and 272 targets were screened using the PubChem and Swiss Target Prediction databases. Introduce it into the software of Cytoscape 3.7.2 and establish the graph of “drug-active ingredient-ingredient target.” A total of 276 AD targets were obtained from OMIM, Gene Cards, and DisGeNET databases. Import the intersection targets of drugs and diseases into STRING database for enrichment analysis, and build PPI network in the Cytoscape 3.7.2 software, whose core targets involve APP, AMPK, NOS3, etc. GO analysis and KEGG analysis showed that there were 195 GO items and 30 AD-related pathways, including Alzheimer's disease pathway, serotonin synapse, estrogen signaling pathway, dopaminergic synapse, and PI3K-Akt signaling pathway. Finally, molecular docking was carried out to verify the binding ability between Acori graminei rhizoma and core genes. Our results predict that Acori graminei rhizoma can treat AD mainly by mediating Alzheimer's signal pathway, thus reducing the production of A*β*, inhibiting the hyperphosphorylation of tau protein, regulating neurotrophic factors, and regulating the activity of kinase to change the function of the receptor.

## 1. Introduction

Alzheimer's disease (AD) is a neurodegenerative disease characterized by a severe decline in cognitive function and memory. The main pathological features were abnormal deposition of A *β* and nerve fiber tangles formed by hyperphosphorylated tau protein accumulation [1, 2]. At present, cholinesterase inhibitors are commonly used to improve the patients' cognitive function with AD [3], but these drugs can only alleviate the disease but cannot reverse the disease's progression. Further studies have shown that the etiology of Alzheimer's disease may be related to a series of complex factors, such as heredity and obesity [4]. In diseases with complex pathogenesis, the study of the “multi-component, multi-target” drug action mechanism plays a prominent role [5]. As a multicomponent and multitarget discipline system, network pharmacology has its unique advantages in studying complex molecular mechanisms [6, 7].

In traditional Chinese medicine, AD is often classified as “dementia” caused by brain marrow deficiency and evil, disturbing brain orifices [8]. The data show that traditional Chinese medicine has used buxu, kaiqiao, dissipation blood stasis, and activating blood circulation to treat AD, and the curative effect is reliable [9, 10]. Acori graminei rhizoma (AGR) is a famous Chinese herbal medicine with a pungent taste and warm nature. It has the effect of Xingshen Yizhi and occupies the first place in the single traditional Chinese medicine for the treat AD. Acori graminei rhizoma is widely used in many fields, such as Alzheimer's disease, epilepsy, amnesia, aphasia caused by stroke, and tinnitus. Furthermore, it has been found that Acori graminei rhizoma can play the role of anti-*β*-amyloid protein (A *β*) deposition, anticentral cholinergic nerve function damage, and antineuronal apoptosis [11, 12]. However, the pharmacological research on the treatment of AD is not perfect. In this paper, the network relationship between active components and targets of Acori graminei rhizoma and AD was analyzed by using the method of network pharmacology to provide a molecular study on the mechanism of action of Acori graminei rhizoma in treating AD.

## 2. Materials and Methods

### 2.1. Screening of Components of Acori Graminei Rhizoma

Acori graminei rhizoma's chemical constituents were obtained by consulting the literature and TCMSP database (http://lsp.nwu.edu.cn/tcmsp.php). In this study, the chemical components of Acori graminei rhizoma were screened based on the pharmacokinetic parameters: absorption, distribution, metabolism, and excretion (ADME). The screening conditions were oral bioavailability (OB) ≥ 30% and drug − like (DL) ≥ 0.18. Additionally, a chemical acting on a specific part of the brain must effectively penetrate the blood-brain barrier (BBB) to reach the brain target. To study the mechanism of action of Acori graminei rhizoma on nervous system diseases, BBB can be used as a screening index, and the threshold value of BBB ≥ −0.30 (it is considered that the molecule has certain permeability) is used as a threshold. Finally, the active components of Acori graminei rhizoma, which are not included, are collected and supplemented by literature.

### 2.2. Acquisition of Component Targets

Import the components into the PubChem database (https://Pubchem.ncbi.nlm.nih.gov/), search and collect the 2D structure of the components, import them into the Swiss Target Prediction database (http://www.swisstargetprediction.ch/), and obtain the component targets. Then, using the target protein name in the Uniprot database (http://www.Uniprot.org), the genes were standardized and sorted out, and finally, possible target genes for the compound's action were downloaded and sorted out.

### 2.3. Acquisition of AD Targets

Search for “Alzheimer's disease” in OMIM database (https://www.omim.org/), CTD database (http://CTD.mdibl.org/), and Gene Cards database (https://www.genecards.org/). Using Excel combined data, the target protein was introduced into the Uniprot database for gene standardization, and coincident genes were removed to collect AD disease genes.

### 2.4. Construction of “Drug-Active Ingredient-Target” Network

The molecular formulas of the Acori graminei rhizoma components were expressed by AGR1-AGRn (AGR is the abbreviation of “Acori graminei rhizoma”). The components and corresponding targets were introduced into the Cytoscape3.7.2 software to construct the “drug-active ingredient-target” network and then analyze it. In the network, the active components and protein targets are represented by a node, and an edge connects the nodes. Acori graminei rhizoma's core targets in AD treatment were analyzed according to the betweenness centrality (BC), closeness centrality (CC), and the median of Degree.

### 2.5. Construction of Intersection Target Network between Drugs and Diseases

The intersection gene was introduced into Wei Shengxin website (http://bioinfogp.cnb.csic.es/tools/Venn/index.html), and the Venn diagram was used to show the common target of AGR and AD. The intersection gene was introduced into the STRING database (https://string-db.org/) and searched under the condition of Homo sapiens. 0.40 and 0.70 were used as the moderate correlation threshold and high correlation threshold of Acori graminei rhizoma. The Node1 and Node2 in the results were introduced into the Cytoscape 3.7.2 software. The topological analysis and calculation were carried out using the Network Analyzer tool to construct the “component-target-disease” network. The core target was predicted according to the median of BC, CC, and Degree value.

### 2.6. GO Analysis and KEGG Analysis

Gene ontology (GO) analysis and Kyoto Encyclopedia of Gene and Genome (KEGG) analysis can provide gene expression data and systematic visualization information of target genes. In this study, GO analysis and KEGG functional annotation and enrichment analysis were carried out by using DAVID 6.8 database (https://david.ncifcrf.gov/). According to “*P* ≤ 0.01” on the Wei Shengxin website (http://www.bioinformatics.com.cn/), significantly enriched biological annotations were screened out according to biological information. GO analysis chart and KEGG analysis bubble chart were drawn to predict the pathway of Acori graminei rhizoma in AD treatment.

### 2.7. Molecular Docking of “Drug Components-Core Targets”

The LEDOCK molecular docking software was used to verify the molecular docking, between Acori graminei rhizoma's active components and the core targets in the network analysis, to explore the binding degree between the active components and the core targets.

## 3. Results

### 3.1. Screening of Active Chemical Constituents of Acori Graminei Rhizoma

In this study, there were a total of 105 chemical constituents of Acori graminei rhizoma in the TCMSP database. Three components were screened and numbered according to the conditions of OB ≥ 30%, DL ≥ 0.18, and BBB ≥ −0.30, including 2′-O-methylisoliquiritigenin, calamendiol, and spathulenol. The molecule ID in the table is the identity information of the component, the name of molecule is the compound name of the ingredient, and the component number represents the coding sorted by the acronym of Acori graminei rhizoma.

**Figure 1:**
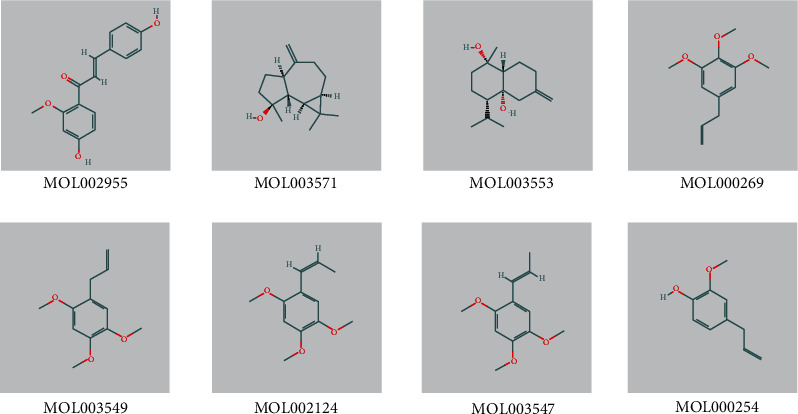
Structure of 8 active components selected from Acori graminei rhizoma.

Modern studies have shown that elemicin, gamma-asarone, *β*-asarone, *α*-asarone, and eugenol are significant constituents in Acori graminei rhizoma, while ADME parameters of the above ingredients are outside the range of screening in the TCMSP database. Our group added them to the list, so there are eight components in total, as shown in Table 1 and Figure 1.

### 3.2. Targets of Drug Ingredients

Imported the above eight components into the PubChem database and Swiss Target Prediction database and obtained the effective component targets of Acori graminei rhizoma. The result included 657 targets, and after removing the repetition value, there remained 272 targets in total.

### 3.3. Targets for AD Disease

Entered “Alzheimer's disease” into the OMIM database, Gene Cards database, and CTD database to get the disease target, where the relevance score or inference is greater than 0. Finally, we combined the database information and deleted the coincident genes. Then, the result consisted of 276 pieces of target information related to AD diseases.

### 3.4. Construction and Analysis of the Drug-Target Network

**Figure 2:**
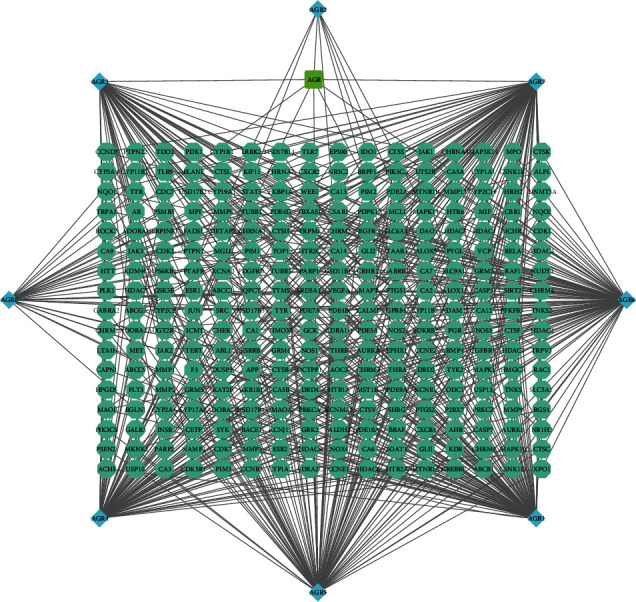
“Drug-active ingredient-component target” network.

We used the Cytoscape 3.7.2 software to construct a PPI network of drug-active ingredient-component targets based on the previous results. The blue diamond represents the active compound of Acori graminei rhizoma, the cyan octagonal represents the target gene, and the green square represents Acori graminei rhizoma, as shown in Figure 2. The network has 281 nodes (1 drug node, eight active component nodes, 272 target nodes, and 577 edges). Network Analyzer calculates the PPI network. The median of BC and CC was 0.00006847 and 0.318182, respectively, and the median of 2 times of Degree value was 4. The core node needs to meet the median card value of the above parameters. The value of *β*-asarone and *α*-asarone is higher, followed by 2′-O-methylisoliquiritigenin and other components. These active components with higher parameters may play a relatively important role in treating AD disease, and these components may be drug docking targets.

### 3.5. Construction and Analysis of “Drug Ingredient-Target-Disease” Network

**Figure 3:**
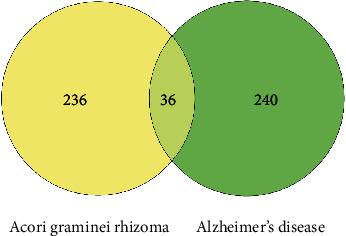
Venn diagram of the overlapping genes of Acori graminei rhizoma and Alzheimer's disease.

The Venn diagram of the intersection target was drawn through the Wei Shengxin website to analyze the intersection target between the component target of Acori graminei rhizoma and AD. As shown in Figure 3, we found 36 intersection targets between the component target of Acori graminei rhizoma and AD, and the common targets were APP, CASP3, MAPK1, ACHE, and others.

**Figure 4:**
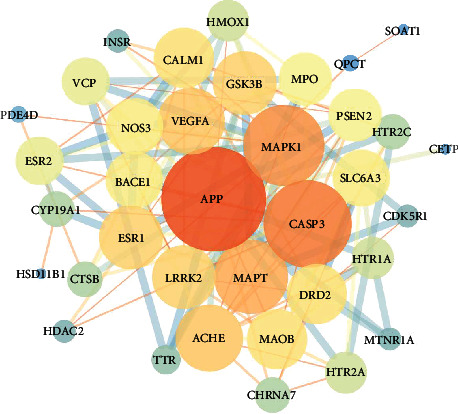
Construction of protein-protein interaction network of Acori graminei rhizoma under 0.4 threshold. 36 nodes represent 36 proteins, and 138 edges represent the interactions between 138 pairs of proteins. The size and color of the node represent the degree, and the size and color of the edge represent the comprehensive score.

**Figure 5:**
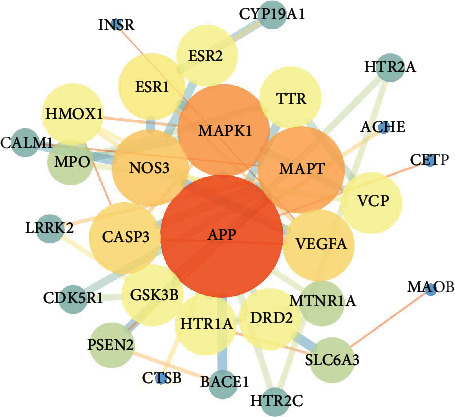
Construction of a protein-protein interaction network expressed by a common target at a threshold of 0.7. 30 nodes represent 30 proteins, 59 edges represent 59 pairs of protein interactions, node size and color represent, and edge size and color represent comprehensive scores.

The purpose of uploading the above 36 targets to the STRING database was to draw the protein-protein interaction (PPI) network between drugs and diseases and then gained the visual analysis through the Cytoscape 3.7.2 software. The size and color of the node are correlated with the Degree value positively. The larger the Degree value, the larger the node and the darker the color, indicating that the target is more important in this network relationship. A PPI network with a threshold of 0.4 is constructed (Figure 4). BC ≥ 0.00819305, CC ≥ 0.493055555, and Degree ≥ 14 (2 times median) are calculated by the Cytoscape 3.7.2 software. The core targets APP, CASP3, MAPK1, MAPT, VEGFA, ACHE, GSK3B, ESR1, LRRK2, DRD2, and so on, which are located in the center of the network and have a high overall score, are selected and used as molecular docking targets (Table 2). To calculate BC ≥ 0.00400246, CC ≥ 0.4084507, and Degree ≥ 6 (2 times the median), we constructed a PPI network with a threshold of 0.7 (Figure 5), calculated the common target genes, and deleted the unconnected nodes in a further study. The network shows that APP, MAPK1, MAPT, NOS3, VEGFA, and CASP3 are situated in the network center. The results of the central target of Acori graminei rhizoma in the treatment of AD disease suggest that it may play an efficient role in the pharmacological action of Acori graminei rhizoma.

### 3.6. Functional Pathway Annotation of Drug Ingredient Targets

**Figure 6:**
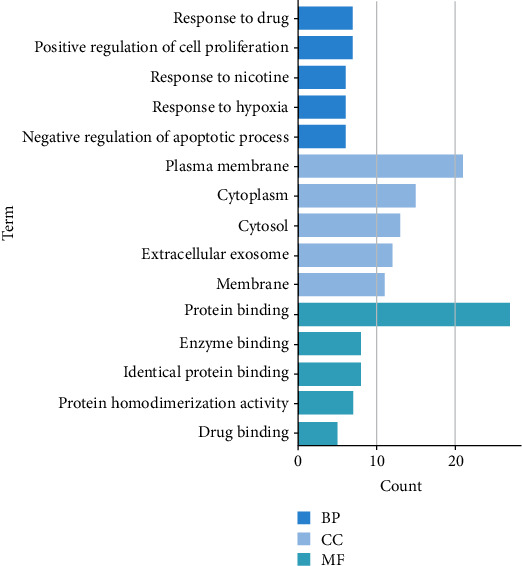
GO analysis results. The results of biological processes show that Acori graminei rhizoma mainly mediates the response of the disease to drugs and participates in signal transduction. The results of cell composition show that the plasma membrane and cytoplasm are the key regions. The results of molecular function study show that binding to protein is the main pathway of action.

In this study, a total of 195 GO items were enriched, and the first 15 GO items with the lowest *P* value were selected for mapping (*P* < 0.05), as shown in Figure 6. The *Y*-axis represents the GO entry, and the area size of the *X*-axis and bar chart represents the number of genes belonging to GO in the target gene set. In the biological process (BP), the typical targets are mainly concentrated in the response to drugs, the positive regulation of cell proliferation, the response to nicotine, the response to hypoxia, the negative regulation of apoptosis, and so on. In the cellular component (CC), the typical targets are mainly concentrated in the plasma membrane, cytoplasm, extracellular body, membrane, etc. In terms of molecular functional (MF), the typical targets are mainly related to protein binding, enzyme binding, same protein binding, protein homodimerization activity, drug binding, and so on.

**Figure 7:**
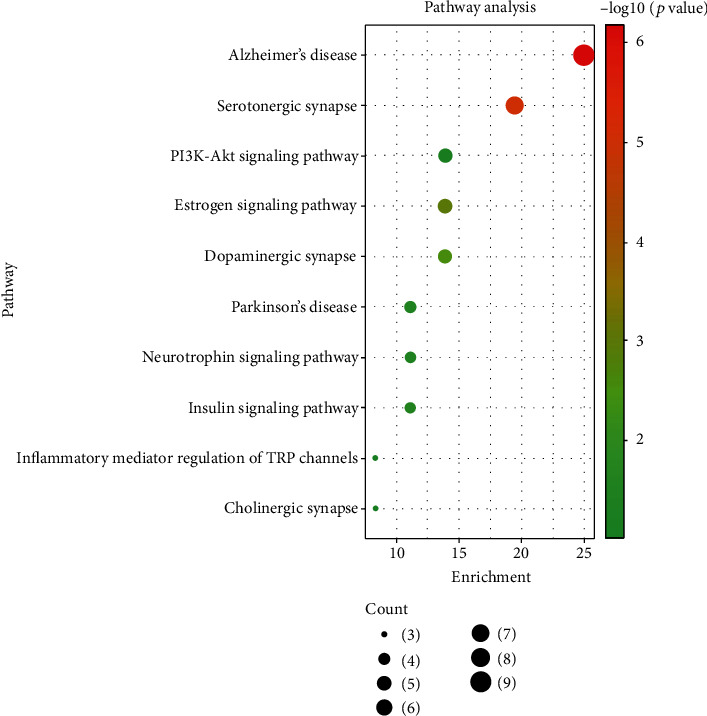
KEGG results show that related pathways include Alzheimer's disease, serotonergic synapses, estrogen signal pathways, dopaminergic synapses, etc.

We obtained a total of 30 enrichment pathways through KEGG pathway enrichment and drew the bubble diagram according to the first ten pathways with the lowest *P* value combined with biological annotations (Figure 7). *Y*-axis represents the name of the pathway, *X*-axis and bubble area represent the number of genes belonging to this signal pathway in the target gene set, and bubble color represents enrichment significance, that is, the size of *P* value. These common targets are enriched in Alzheimer's disease, serotonergic synapses, HIF-1 signal pathways, estrogen signaling pathways, alcoholism, cocaine addiction, dopaminergic synapses, prolactin signal pathways, gap junctions, and neuroactive ligand-receptor interactions. The analysis of this series of practical tests can provide valuable information to explain the possible mechanism of Acori graminei rhizoma in AD treatment.

### 3.7. Results of “Drug Component-Core Target” Molecule Docking

**Figure 8:**
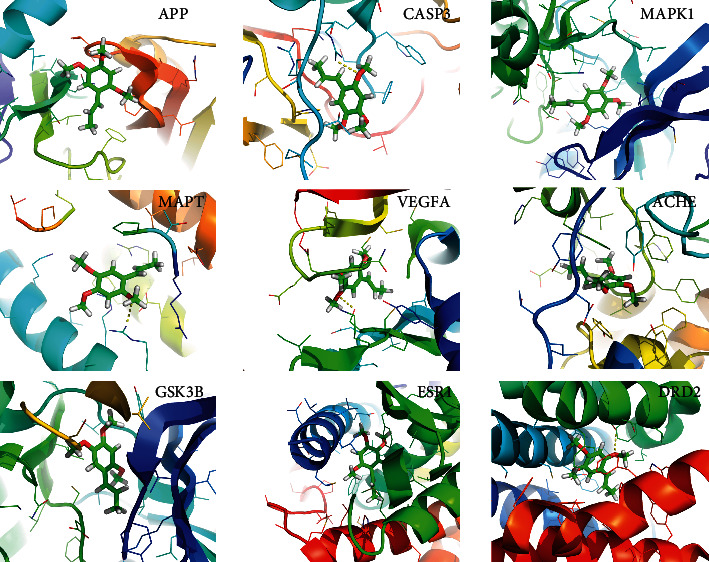
Molecular docking diagram of *β*-asarone and core target.

The study used ten core targets with a high score in protein-protein interaction for molecular docking. Since there was no data on LRRK2, the rest nine core targets were selected to conduct molecular docking with Acori graminei rhizoma. The docking score is shown in Table 3. It is generally believed that a score of less than 5 kJ/mol indicates a good binding activity between the compound and the target. The molecular docking results show that the score of core compounds (*β*-asarone, *α*-asarone, 2′-O-methylisoliquiritigenin, gamma-asarone) and core targets (APP, CASP3, MAPK1, ACHE) is less than 5 kJ mol^−1^, indicating that the core compounds have good binding activity with core targets. The docking mode between *β*-asarone and nine core targets is shown in Figure 8.

## 4. Discussion

Studies have shown that among the single drugs of traditional Chinese medicine in treating Alzheimer's disease, Acori graminei rhizoma appears with the highest frequency [13, 14], so we choose Acori graminei rhizoma as the research object, which has a significant clinical application value. Eight candidate active components such as *α*-asarone, *β*-asarone, and eugenol in Acori graminei rhizoma were screened in this study. The “drug-active ingredient-target” network diagram was constructed by the Cytoscape 3.7.2 software, and it was found that a complex interaction between components and target. The PPI network of “component-target-disease” is constructed based on the STRING database. According to the topology parameters, the factors with high scores are APP, CASP3, MAPK1, MAPT, VEGFA, ACHE, GSK3B, ESR1, LRRK2, DRD2, and so on. A close relationship exists between amyloid precursor protein (APP) and *β*-amyloid protein (A *β*) accumulation. APP produces A *β* through secretase cleavage. Excessive secretion and accumulation of A *β* in the brain will cause cytotoxicity and injury, cause an inflammatory reaction, and finally lead to nerve cell apoptosis and degenerative lesions [15–17]. Caspase-3, as a highly enriched molecule in PPI analysis, can directly and effectively cleave APP to the direction of A *β* formation [18]. Interestingly, acetylcholinesterase (AChE) can not only hydrolyze acetylcholine (ACh) but also accelerate A *β* aggregation and amyloid fibril formation [19]. The D2 receptor in the dopamine-related pathway is closely related to the abnormal accumulation of tau protein. Clinical use of D2 receptor antagonists can reduce the concentration of insoluble tau protein and neurotoxicity [20]. The change of glycogen synthesis kinase (GSK3 *β*) activity affects tau hyperphosphorylation and participates in AD's formation and development [21]. The activity of GSK3 *β* is regulated by the classical antiapoptotic signal pathway phosphatidylinositol 3-kinase/protein kinase B (PI3K/AKT) pathway [22–24]. At the same time, mitogen-activated protein kinase (MAPKs) is another important pathway to regulate cell growth and apoptosis, in which the p38MAPK pathway is also related to the pathogenesis of AD. The activation of p38MAPK leads to hyperphosphorylation of tau, which leads to neurofibril entanglement. p38MAPK accelerates the course of AD by producing neurotoxic proinflammatory factors, increasing A *β* deposition, and activating caspase-3 to induce apoptosis of hippocampal neurons [25].

In this study, 10 key targets of Acori graminei rhizoma in treating AD were obtained, but the LRRK2 lacked relevant data so that the rest nine targets were selected for molecular docking with drugs. The molecular docking of 2′-O-methylisoglycine to the core target is very strong, which suggests that it has a research prospect. In molecular docking, we found that *β*-asarone has a strong binding activity with APP, and it also has a good performance in the docking diagram. In the process of consulting the relevant literature, we found that *β*-asarone can indeed affect the related targets of AD, which suggests that *β*-asarone may be a key component in treating AD. *β*-asarone may reduce the production of A *β* and reduce the toxic damage of A *β* to neurons and synaptic ultrastructure by inhibiting the overexpression of APP or promoting the decomposition and excretion of APP [26]. At the same time, *β*-asarone can inhibit the phosphorylation of JNK in hippocampal neurons, upregulate the expression of Bcl-2 protein, and downregulate the expression of caspase-3 at the transcriptional level, thus play a role in antiapoptosis of hippocampal neurons [27]. Other studies have shown that *β*-asarone in the volatile oil of Acori graminei rhizoma can restore the phosphorylation level of GSK-3 *β* and activate Wnt/*β*-catenin pathway, thus reducing AD caused by tau hyperphosphorylation and A *β* accumulation [28]. Furthermore, *β*-asarone has the effect of acetylcholine inhibitor to reduce AChE and can inhibit the production of A *β* 42 [29], which can alleviate the AD's development. Interestingly, other components of Acori graminei rhizoma also play a specific role in interfering with AD. Eugenol in the component inhibits NF-*κ*B and MAPK pathway and has antiapoptosis and antioxidant activities and may also play a role in treating AD [30, 31]. *α*-Asarone can protect glial cells by inhibiting the release of inflammatory cytokines [32, 33].

To explain the selected key targets' role in gene function and signal pathway, GO functional enrichment analysis and KEGG pathway enrichment analysis were carried out in this study. According to the analysis results, some common targets are enriched in Alzheimer's disease, serotonergic synapses, HIF-1 signal pathways, estrogen signal pathways, dopaminergic synapses, neuroactive ligand-receptor interactions, and so on. Serotonin can maintain nerve excitability. When its expression decreased, it will aggravate brain neuron damage and clinical manifestation of cognitive impairment [34]. Eugenol in Acori graminei rhizoma can normalize the concentration of 5-hydroxytryptamine in the brain [35], which helps recover nerve injury and improve AD symptoms. In the treatment of *β*-asarone combined with L-dopa, it was found that *β*-asarone may reduce dopaminergic neurons' damage by regulating the HSP70/MEF2D/Beclin1 pathway in rats [36]. It corresponds to the dopaminergic synapses in KEGG analysis, and the dopamine system plays an efficient role in the pathological process of AD.

**Figure 9:**
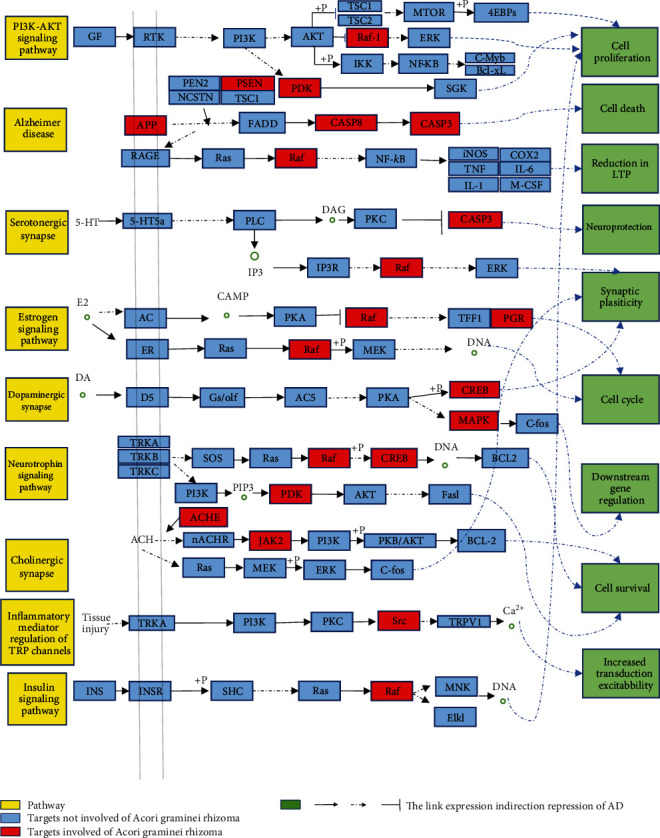
Hypothetical mechanism of Acori graminei rhizoma in the treatment of AD (the yellow box is the signal pathway, the red box is the target of the direct action of the drug, and the green box is the participating biological process).

Interestingly, only moderate dopamine signals can optimize the cognitive function of brain [37]. Estrogen receptor (ESR1) is one of our group's core targets [38], and the estrogen pathway is one of the crucial pathways obtained from enrichment analysis. Studies have shown that estrogen has a neuroprotective effect on AD [39], but the expression of estrogen or ESR1 in AD is downregulated [40]. *β*-Asarone, one of the main components of Acori graminei rhizoma, decreased the concentration of calcium, inhibited apoptosis, and alleviated the dementia damage of vascular endothelial cells induced by *β*-amyloid protein in the AD cell model [41]. Studies have shown that ESR1 may participate in AD's pathophysiology by regulating the transport of calcium ions. However, there are no specific studies to show whether Acori graminei rhizoma can regulate calcium concentration through the estrogen pathway to restore normal brain function by affecting ESR1. It needs further exploration. In order to facilitate the discussion and analysis of the mechanism of Acorus tatarinowii in the treatment of Alzheimer's disease, our research team selected the more relevant signal pathways, action targets, and biological processes and made a hypothetical mechanism diagram (Figure 9).

## 5. Conclusion

To sum up, in this study, the network pharmacology method was used to analyze the complex network relationship between multicomponents as well as multitargets of Acori graminei rhizoma and AD disease targets, which was to explore the molecular mechanism of Acori graminei rhizoma in the treatment of AD. As a result, we found prominent signal molecules such as APP, CASP3, and MAPK1, as well as key signal pathways like Alzheimer's disease and serotonergic synapses. Among them, pathways, for instance, alcoholism and cocaine addiction, are closely related to the pathogenesis, suggesting that these potential pathways are worth studying. At the same time, we predict that the main components of Acori graminei rhizoma in treating of AD are *α*-asarone and *β*-asarone. The therapeutic effect of Acori graminei rhizoma on AD has been carried out clinically, and the curative effect is remarkable. On this basis, this study preliminarily verified the pharmacological mechanism of Acori graminei rhizoma in the treatment of AD, which laid a foundation for further research from the level of cell biology.

## Figures and Tables

**Table 1:** Information of 8 active chemical constituents selected from Acori graminei rhizoma.

Molecule ID	Molecule name	Component number	OB	DL	BBB
MOL002955	2′-O-Methylisoliquiritigenin	AGR1	75.86	0.62	-0.16
MOL003571	Spathulenol	AGR2	81.61	0.78	1.55
MOL003553	Calamendiol	AGR3	61.13	0.18	0.67
MOL000269	Elemicin	AGR4	21.94	0.06	1.28
MOL003549	Gamma-asarone	AGR5	22.76	0.06	1.33
MOL002124	*β*-Asarone	AGR6	35.61	0.06	1.24
MOL003547	*α*-Asarone	AGR7	38.39	0.06	1.18
MOL000254	Eugenol	AGR8	56.24	0.04	1.32

**Table 2:** Topological values of core targets in protein-protein interaction networks.

Core target	Betweenness centrality	Closeness centrality	Degree
APP	0.36432506	0.72916667	23
CASP3	0.12727436	0.66037736	18
MAPK1	0.10814869	0.625	16
MAPT	0.05142191	0.60344828	14
VEGFA	0.05260249	0.58333333	12
ACHE	0.03699421	0.56451613	12
GSK3B	0.02680704	0.57377049	11
ESR1	0.04004646	0.58333333	11
LRRK2	0.01789274	0.56451613	11
DRD2	0.03274326	0.53030303	10

**Table 3:** Results of molecular docking of key targets and their corresponding compounds.

Serial number	Core target	Compound	LeDock score
1	APP	*α*-Asarone	-11.83 kJ/mol
2		*β*-Asarone	-11.58 kJ/mol
3		2′-O-Methylisoliquiritigenin	15.72 kJ/mol
4	CASP3	*α*-Asarone	-14.00 kJ/mol
5		*β*-Asarone	-14.04 kJ/mol
6		2′-O-Methylisoliquiritigenin	-24.45 kJ/mol
7	MAPK1	*α*-Asarone	-14.63 kJ/mol
8		*β*-Asarone	-15.05 kJ/mol
9		2′-O-Methylisoliquiritigenin	-23.12 kJ/mol
10	MAPT	*α*-Asarone	-14.55 kJ/mol
11		*β*-Asarone	-14.63 kJ/mol
12		2′-O-Methylisoliquiritigenin	-23.24 kJ/mol
13	VEGFA	*α*-Asarone	-14.80 kJ/mol
14		*β*-Asarone	-14.80 kJ/mol
15		2′-O-Methylisoliquiritigenin	-19.31 kJ/mol
16	ACHE	*α*-Asarone	-15.51 kJ/mol
17		*β*-Asarone	-15.59 kJ/mol
18		2′-O-Methylisoliquiritigenin	-24.70 kJ/mol
19	GSK3B	*α*-Asarone	-13.54 kJ/mol
20		*β*-Asarone	-13.60 kJ/mol
21		2′-O-Methylisoliquiritigenin	-22.74 kJ/mol
22	ESR1	*α*-Asarone	-15.35 kJ/mol
23		*β*-Asarone	-15.38 kJ/mol
24		2′-O-Methylisoliquiritigenin	-23.49 kJ/mol
25	DRD2	*α*-Asarone	-14.84 kJ/mol
26		*β*-Asarone	-15.17 kJ/mol
27		2′-O-Methylisoliquiritigenin	-21.57 kJ/mol

## Data Availability

The data used to support the findings of this study are included within the article.
